# Altered Behavior in Mice Socially Isolated During Adolescence Corresponds With Immature Dendritic Spine Morphology and Impaired Plasticity in the Prefrontal Cortex

**DOI:** 10.3389/fnbeh.2018.00087

**Published:** 2018-05-09

**Authors:** William E. Medendorp, Eric D. Petersen, Akash Pal, Lina-Marie Wagner, Alexzander R. Myers, Ute Hochgeschwender, Kenneth A. Jenrow

**Affiliations:** ^1^Neuroscience Program, Central Michigan University, Mount Pleasant, MI, United States; ^2^College of Medicine, Central Michigan University, Mount Pleasant, MI, United States; ^3^Department of Psychology, Central Michigan University, Mount Pleasant, MI, United States

**Keywords:** neuronal plasticity, social isolation, social interaction, dendritic spines, long term potentiation, prefrontal cortex

## Abstract

Mice socially isolated during adolescence exhibit behaviors of anxiety, depression and impaired social interaction. Although these behaviors are well documented, very little is known about the associated neurobiological changes that accompany these behaviors. It has been hypothesized that social isolation during adolescence alters the development of the prefrontal cortex, based on similar behavioral abnormalities observed in isolated mice and those with disruption of this structure. To establish relationships between behavior and underlying neurobiological changes in the prefrontal cortex, Thy-1-GFP mice were isolated from weaning until adulthood and compared to group-housed littermates regarding behavior, electrophysiological activity and dendritic morphology. Results indicate an immaturity of dendritic spines in single housed animals, with dendritic spines appearing smaller and thinner. Single housed mice additionally show impaired plasticity through measures of long-term potentiation. Together these findings suggest an altered development and impairment of the prefrontal cortex of these animals underlying their behavioral characteristics.

## Introduction

Socially isolated mice have been used as a model for several psychiatric phenotypes, including depressive-like behavior (Koike et al., 2009; Amiri et al., 2015b), social deficits (Koike et al., 2009; Okada et al., 2015) and anxiety behaviors (Ago et al., 2007; Amiri et al., 2015b). In addition to these abnormal behaviors, these animals exhibit alterations in neurotransmission (Haj-Mirzaian et al., 2015; Okada et al., 2015), and demonstrate abnormal pharmacological responses (Ago et al., 2007; Koike et al., 2009; Amiri et al., 2015a). These behaviors among socially isolated mice have been shown to be improved with treatment of methylphenidate, a common treatment for Attention Deficit Hyperactivity Disorder (ADHD), and with fluoxetine, a selective serotonin reuptake inhibitor, leading some to suggest socially isolated mice as a potential model for studying the pathophysiology of psychiatric disorders of ADHD and depression (Koike et al., 2009). The aberrant social behavior exhibited by socially isolated mice have led some to suggest them as a potential model for autism (Balemans et al., 2010; Reser, 2014; Wöhr, 2015).

Descriptions of and research into socially isolated animals has focused primarily on behavioral differences (Ago et al., 2007; Amiri et al., 2015a; Haj-Mirzaian et al., 2015). Although the behavior provides a clear phenotypic difference as a result of social isolation, no study has provided an understanding of the underlying neurobiological mechanisms that contribute to the emergence of such behaviors. It is likely that a loss of sensory input from social interaction results in underdevelopment within select regions (Harada et al., 2007; Ackman and Crair, 2014), similar to disruptions to ocular dominance seen within the visual system when the eye is occluded (Frenkel and Bear, 2004; Hensch, 2005). These effects have been demonstrated in more complex circuits, such as the basolateral amygdala-prefrontal cortex bidirectional pathway (Castillo-Gómez et al., 2017; Ueno et al., 2017), which are areas that are correspondingly underdeveloped in humans with socio-emotional deprivation (Eluvathingal et al., 2006).

The prefrontal cortex is involved in many advanced cognitive tasks and goal-directed behaviors (Miller, 2000; Buschman and Miller, 2014), and has been demonstrated to be a center for emotional regulation and modulation of social behaviors (Groenewegen and Uylings, 2000; Séjourné et al., 2015; Liu et al., 2016). Dysregulation of neural activity in this area is associated with a variety of psychiatric phenotypes including anxiety, depressive-like behaviors, and social dysfunction (Yizhar et al., 2011; Chaudhury et al., 2013; Saitoh et al., 2014; Suzuki et al., 2016). Development of the prefrontal cortex continues well into early adulthood (Bossong and Niesink, 2010), with many essential alterations during adolescence (Tseng and O’Donnell, 2007; Bossong and Niesink, 2010). Social behaviors begin to emerge during this critical developmental period (Bossong and Niesink, 2010), chief among them being social play in rodents among peers (Vanderschuren et al., 1997, 2016). This evidence suggests social interaction during this critical development stage is likely necessary for normalized social behavior in adult rodents.

The infralimbic nucleus of the prefrontal cortex has been shown to regulate social behavior, and overactivation of this region can induce social deficits in mice (Yizhar et al., 2011). This region receives inputs from the basolateral amygdala and activation of the amygdaloid projections to the prefrontal cortex has been shown to undergo Long Term Potentiation (LTP; Maroun and Richter-Levin, 2003). Acute stress inhibits LTP in this circuit in rats (Maroun and Richter-Levin, 2003). Social isolation is stressful for animals (Zlatković et al., 2014; Fox et al., 2015), and has been shown to affect morphology of inhibitory circuits in the prefrontal cortex infralimbic area and basolateral amygdala (Castillo-Gómez et al., 2017). Although altered morphology has been described, no functional measure has been documented using electrophysiological methods.

Contribution of social isolation to behavioral dysfunction such as anxiety- or depressive-like behaviors, is thought to be related to modulations to the underlying neurological function of the prefrontal cortex in rodents (Koike et al., 2009; Browne et al., 2016; Liu et al., 2016). A lack of social enrichment during this critical period of adolescence likely removes necessary stimulation from the region, giving rise to impaired development of neural connectivity and structure. Reduced stimulation during a sensitive period can reduce the activity within the region, resulting in fewer synaptic changes (Hensch, 2005; Morishita and Hensch, 2008). This can be assessed through dendritic spine morphology and electrophysiological methods to assess plasticity. Spine morphology can act as a marker of this plasticity, with smaller, thinner dendritic spines demonstrating a developmental immaturity (Hayashi and Majewska, 2005; Tanaka et al., 2008). LTP can be directly measured through electrophysiological methods, verifying whether any visible morphological changes in dendritic spines can be directly associated with changes in plasticity. Thus, this research will examine the relationship of the abnormal behaviors found in socially isolated animals and aspects of the functional development of the prefrontal cortex.

## Materials and Methods

### Animals/Genotyping

All experiments involving animals were carried out following the guidelines and protocols approved by the Institutional Animal Care and Use Committee at Central Michigan University and were in compliance with the US National Research Council’s Guide for the Care and Use of Laboratory Animals, the US Public Health Service’s Policy on Humane Care and Use of Laboratory Animals, and Guide for the Care and Use of Laboratory Animals.

Thirty-two Thy-1 GFP^+/–^ mice, line M, were used for testing (Feng et al., 2000; Jackson Laboratories Stock Number 007788^1^, RRID:IMSR_JAX:007788). Thy-1 mice express green fluorescent protein sparsely throughout the cortex (Kumar et al., 2013; Keifer et al., 2015; Vandenberg et al., 2015), allowing for imaging of single neurons within the prefrontal cortex pyramidal neurons. The heterozygous offspring were weaned at 21 days and segregated into either single housed or group housed cages. Mice remained in those cages for 3 months. At 4 months of age, mice were tested behaviorally, then either perfused for confocal imaging or used for electrophysiology. Mice were housed in ventilated cages under 12-h reverse light cycle and allowed to feed *ad libitum*. Mice were moved between holding room and surgical or behavioral suite, located within the same facility. Behavioral tests were carried out during the day in rooms under reverse light cycle.

Genotypes were determined by PCR using primers from the Thy-1 sequence (forward primer 5′-AGACACAC ACCCAGGACATAG) and the EGFP sequence (reverse primer 5′-GGTGGTGCAGATGAACTTCAG). PCR reactions were performed in 25 μL total volume containing 10 mM dNTP, 10 μM forward and reverse primer, 1 unit Taq polymerase, with 1 μL tail DNA. Mouse tail DNA was prepared by digesting overnight in 55°C in 100 μL buffer containing proteinase K (Laird et al., 1991).

### Three Chamber Test

Social behavior was tested using the 3-chamber test (Crawley, 2007; Yang et al., 2011). Animals were placed in a 27″ × 14″ chamber with three segments of equal size. Animals were allowed to explore the arena for 5 min to habituate to the apparatus. Mice were tested first for social approach, which has been demonstrated to relate to social deficits found in autism (Yang et al., 2011). Mice were additionally tested for social novelty immediately following the social approach test.

#### Social Approach

**Figure 1:**
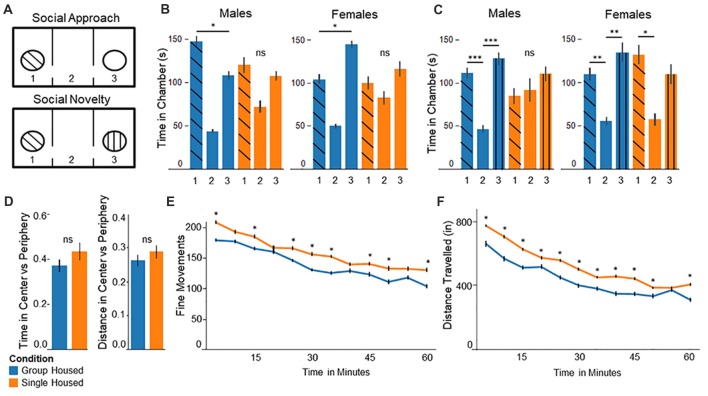
Behavior testing confirms abnormal behaviors among socially isolated mice. **(A)** Three chamber test experimental setup: *(upper panel)* In the social approach test, the stranger mouse is placed in one of the chambers and the other left empty. *(lower panel)* In the social novelty test the previously empty chamber is filled with a novel stranger mouse. **(B)** Social Approach Test: *(left)* Group housed males show significantly more time in chamber 1, interacting with the stranger mouse. Single housed males show no significant differences between chambers. *(right)* Group housed females spend significantly more time in the empty chamber 3. Single housed females show no significant differences between chambers. **(C)** Social Novelty Test: *(left)* Group housed males show significantly more time in the outer chambers compared to the center, empty chamber. Single housed males show no significant differences between chambers. *(right)* Group housed females spend significantly more time in chamber 3 compared to the center chamber. Single housed females show significantly more time spent in chamber 1, with the familiar mouse. **(D)** In the open field test single housed mice show no significant differences on either distance traveled or total time spent in the center vs. the periphery compared to group housed mice. **(E)** Single housed mice show significantly increased fine movements compared to group housed animals. **(F)** Single housed mice show significantly increased distance traveled compared to the group housed animals, suggesting some hyperlocomotion. *Bonferroni post hoc* test: *p < 0.05, **p < 0.01, ***p < 0.001. ns, non-significant.

Two identical plastic cylinders were placed in either of the external chambers. These cylinders were clear, with regular holes to allow for visual and olfactory stimuli. A sex-matched, non-familiar mouse was placed in one of these cylinders (See Figure 1A, upper panel). Experimental mice were placed in the middle, empty section of the 3-chamber apparatus and allowed to roam for 5 min. Social behavior was characterized by time spent in the chamber containing the mouse, compared to the empty chamber.

#### Social Novelty

Following the social approach test, a novel, sex-matched, non-familiar mouse was placed in the previously empty cylinder (See Figure 1A, lower panel). Experimental mice were again allowed to roam for 5 min. Social behavior was characterized by time spent in the chamber containing the novel mouse compared to the previous mouse. The chamber was cleaned with 70% ethanol between testing mice.

### Open Field

Experimental mice were placed in a 17″ × 9″ cage and allowed to roam for 60 min. Movements were tracked by laser grid and analyzed using Hamilton-Kinder™ Motor Monitor software. Overall ambulation of the mice was assessed to establish normal exploratory behavior. Time spent in the center vs. periphery was used to assess for anxiety behaviors, with increased time in the center as evidence of less anxiety (Crawley, 1985). Data was plotted in 5 min intervals.

### Forced Swim

Mice were placed in a 5″ wide cylinder filled with water. Mice were allowed to swim for 6 min. The first 2 min are considered habituation and removed from analysis. Movements within the cylinder were tracked using Hamilton-Kinder™ motor monitor software. Depressive-like behavior was characterized by reduced overall beam breaks (immobility) and increased total rest time. This test was repeated a second day, since evidence has shown socially isolated mice may differ on later trials, demonstrate reduced immobility (less depressive-like behavior) compared to group housed mice in initial trials, and demonstrating a substantially increased immobility (increased depressive-like behavior) in subsequent trials (Koike et al., 2009).

### Confocal Imaging

At 4 months of age mice were perfused using 4% paraformaldehyde (PFA) and their brains were removed. Brains were post-fixed in 4% PFA overnight. Brains were then transferred to 30% sucrose solution to dehydrate for 3 days or until they no longer float in the solution. After brains were fully dehydrated, they were dropped in 2-methyl butane cooled with dry ice to flash freeze the tissue, then transferred to −80°C for storage.

Brains were sectioned on a Microtome^®^ cryostat at 200 μm thickness. Sections began at the olfactory bulbs through 15, 200 μm sections for a total of 3 mm, ensuring full sectioning of the prefrontal cortex. Sections were mounted onto slides and cover-slipped using Aqua-Mount™. Slides were then sealed using clear nail polish to limit oxygenation and deterioration of the tissue. Slides were stored at 4°C in the dark until imaging.

Images were taken with an Olympus™ Confocal Microscope at 60× using an oil immersion lens at 2048 × 2048 pixels with 0.25 μm between images. Z-stacks vary between 88 and 169 depending on the depth of the dendritic arborization. Upper and lower bounds were set as the highest and lowest points of the dendritic arbors from the region of interest. Images were processed and quantified using Neurolucida^©^ neuron tracing software (RRID:SCR_001775). Dendrites were traced using semi-automated tracing. Spines were identified using automated identification with 0.4 μm minimum length and 4.0 μm maximum length, as well as filtering noise.

### Electrophysiology

Mice underwent surgical placement of two electrodes under isofluorane anesthesia. A bipolar electrode was placed in the amygdala, and a tri-lead, monopolar electrode was placed in the medial prefrontal cortex. This allows stimulation of the amygdala, which has reciprocal connections to the prefrontal cortex (Cassell et al., 1989; Amaral and Insausti, 1992). The amygdala electrode was placed at 1.58 mm posterior to bregma, 3.35 mm lateral to the midline and 4.15 mm ventral to the cranial surface. The medial prefrontal cortex electrode (mPFC) was placed at 1.94 mm anterior to bregma, 0.35 mm lateral to the midline and 2.20 mm ventral to the cranial surface. The tripolar mPFC electrode had two monopolar recording leads, with a 0.5 mm vertical offset between them. Recordings were taken from the monopolar lead with the greatest evoked potential. Three machine screws (Plastics One) were placed on either side of the electrode barrels for stability. The third lead from the tri-lead electrode was grounded to a machine screw and locked in place using adhesive silver epoxy (MG Chemical). Both electrodes were cemented in place with dental acrylic (Ortho-Jet, Lang Dental).

Electrodes were set in place with the mouse in a stereotaxic frame. During surgery, electrodes were recorded from *in vivo*. Evoked potentials were recorded from the mPFC while delivering a stimulation of 1000 μA to the amygdala. Electrodes were moved ventrally until evoked potentials were at a peak or until target depth was reached.

### Long-Term Potentiation Stimulation

Mice were anesthetized with a 1 g/kg injection of urethane before stimulation. An input/output curve was measured for each mouse by delivering a test-pulse stimulation to the amygdala beginning at 50 μA–1500 μA in stepwise increases of 100 μA. Five evoked potentials were recorded from each stimulation level. Responses were examined to determine a saturation level. A test-pulse amplitude level was chosen roughly 200 μA below the saturation level. Evoked potentials were then recorded every 30 s for a total of 10 min. Upon completion of these 10 min, mice were delivered a set of three, 10-train bursts at 100 Hz. A supraphysiological theta-burst stimulation was delivered in the form of five train bursts per second over 2 s. Bursts were each separated by 1 min intervals to induce LTP. Upon induction of LTP, evoked potentials were again recorded for a total of 60 min.

### Experimental Design and Statistical Analysis

For each behavioral experiment, a total of 32 mice were used, with 16 mice socially isolated and 16 mice group housed (8 male, 8 female per group). For confocal experiments, eight brains were used, four from each group with 2 males and 2 females in each. Each brain processed included between 13 and 23 dendritic arbor segments (average 17). Electrophysiological experiments included eight animals, four animals from each group (2 male, 2 female). Male and female mice were grouped where no sex differences were found. All data are expressed as mean ± SEM. Analysis was conducted in Statistical Package for Social Statistics (SPSS) software (IBM, RRID:SCR_002865).

#### Three Chamber Test

One-way analysis of variance (ANOVA) was run on time between chambers with Tukey’s *post hoc* tests to test for significant differences.

#### Open-Field Test

Three-Way Repeated Measures ANOVA was run by housing condition, by sex and by time interval (5 min intervals over 60 min). Tukey’s *post hoc* tests were run where significant main effects were found.

#### Forced-Swim Test

Three-Way Repeated Measures ANOVA was run by housing condition, by sex and by trial. Tukey’s *post hoc* tests were run where significant main effects were found.

#### Confocal Imaging

Two-way ANOVA was run by housing condition and by sex. Tukey’s *post hoc* tests were run where significant main effects were found.

#### Electrophysiology

Two-way ANOVA was run by housing condition and by sex. Tukey’s *post hoc* tests were run where significant main effects were found.

## Results

### Socially Isolated Animals Show Altered Social Behavior in the Three-Chamber Test

In the social approach task animals socially isolated during adolescence display abnormal social behavior, replicating results identified by others. Group housed males spent significantly more time in chamber 1 with the interaction mouse, compared to the empty chamber 3 (*F*_(2,21)_ = 27.41, *p* = 1.0 × 10^−6^, Figure 1B, left). Conversely, single housed males show no significant differences on time spent between chambers (*F*_(2,21)_ = 2.77, *p* = 0.09), demonstrating abnormal social behavior. Surprisingly, group housed females show significantly more time spent in the empty chamber 3, compared to chamber 1 with the interaction mouse (*F*_(2,21)_ = 33.79, *p* = 2.7 × 10^−7^, Figure 1B, right). Socially isolated females show no significant differences between chambers (*F*_(2,21)_ = 1.03, *p* = 0.37), again demonstrating abnormal social behavior among socially isolated animals.

In the Social Novelty task single housed mice show impaired sociability. Group housed mice show significant differences on time spent between chambers, with significantly more time spent in the outer chambers compared to the center, empty chamber (*F*_(2,21)_ = 11.85, *p* = 0.0004; Figure 1C, left). By contrast, single housed males show no significant differences (*F*_(2,21)_ = 0.39, *p* = 0.68). Group housed females behave more as expected here, with significantly more time spent in chamber 3 with the novel mouse, compared to the center chamber (*F*_(2,21)_ = 5.93, *p* = 0.009; Figure 1C, right). Single housed females show significant differences between chambers; however, they show significantly more time spent in chamber 1, with the familiar mouse, compared to the center chamber (*F*_(2,21)_ = 3.66, *p* = 0.04). Together these results suggest abnormal social behavior among single housed animals, demonstrating a lack of preference for the presence of a novel mouse.

### Single Housed Mice Show Hyperlocomotive Behaviors in Open Field

Single housed mice show no significant differences from group housed mice for anxiety (Figure 1D). Single housed mice and group housed mice show similar time spent in the center compared to the periphery, as well as distance traveled in the center vs. periphery. Single housed mice, however, show significantly greater fine movements compared to group housed animals (Two-Way Repeated Measures ANOVA—Condition: *F*_(1,29)_ = 4.35, *p* = 0.045; Time: *F*_(11,319)_ = 35.01, *p* = 1.5 × 10^−48^; Figure 1E). Fine movements are defined as movements in the Z-axis without movement in the X-Y plane, such as rearing behaviors. No sex differences were found on any open field outcomes.

Due to the differences in fine movements, total distance was assessed to further investigate exploratory behaviors of single housed animals. Single housed animals show significantly greater distance traveled compared to group housed animals. The data indicate a significant main effect for housing condition, a significant main effect for time, and a significant interaction effect for condition by time (Two-Way Repeated Measures ANOVA—Condition: *F*_(1,29)_ = 4.45, *p* = 0.044; Time: *F*_(11,319)_ = 93.66, *p* = 6.4 × 10^−93^; Interaction of Condition × Time: *F*_(11,319)_ = 1.96, *p* = 0.032; Figure 1F). Together these data suggest single housed mice exhibit hyperlocomotive behaviors, which has recently been documented by others (Castillo-Gómez et al., 2017).

Although single housed animals display significantly increased fine movements, these fine movements are not well defined. In order to assess what behaviors single housed animals engage in during “fine movements”, animals were placed in an open container and observed for 60 min. Their behaviors during what would be classified as fine movements was categorized into four primary behavior types: (1) sitting, where the mice do not appear to be engaging in any form of movement; (2) smelling, where mice are examining surroundings with visible movements of the head; (3) rearing; and (4) grooming. Data for each category are given as percent of total time engaged in fine movements (Table 1). The data show single housed mice engage in very little grooming behaviors (6.3% compared to 15.7%), and show much more time spent engaging in very little movement (Sitting: 32.5% compared to 22.2%). These data correspond to findings by Haj-Mirzaian et al. (2015) where it was reported that single housed mice, different from group housed mice, engage in significantly reduced grooming behaviors when covered in a 10% sucrose solution.

**Table 1:** Single housed mice show reduced grooming behaviors.

	Sitting	Smelling	Rearing	Grooming
Single	32.5%	35.3%	25.9%	6.3%
Group	22.2%	37.4%	24.7%	15.7%

*Mice were observed for a 1 h period for the behaviors detailed. Behaviors are displayed as percentage of the total time spent engaging in these behaviors*.

### Single Housed Mice Display Some Depressive-Like Behaviors in the Forced Swim Test

The forced swim test replicated findings by Koike et al. (2009), who have demonstrated single housed mice significantly increase in total rest time over multiple trials. Accordingly, the test was performed in two separate trials on separate days. Single housed mice initially show no significant differences compared to group housed animals (*F*_(1,29)_ = 2.30, *p* = 0.14); however, there was a significant main effect for trial for both overall movements and immobility time (Basic Movements: *F*_(1,29)_ = 10.62, *p* = 0.0029; Immobility Time: *F*_(1,29)_ = 7.52, *p* = 0.01, Data Not Shown). *Post hoc* tests reveal single housed mice show a significant decrease in basic movements from Trial 1 to Trial 2 (Tukey’s *post hoc*: *p* = 0.05). Group housed mice show no significant change between trials. Single housed mice additionally show significantly increased immobility time from Trial 1 to Trial 2 (Tukey’s *post hoc*: *p* = 0.05), while, again, group housed mice show no differences between trials. No sex differences were found (*F*_(1,29)_ = 0.70, *p* = 0.41). This confirms previous findings by Koike et al. (2009) demonstrating socially isolated mice are susceptible to depressive-like behaviors.

### Confocal Microscopy Reveals Immature Spine Morphology Among Isolated Mice

**Figure 2:**
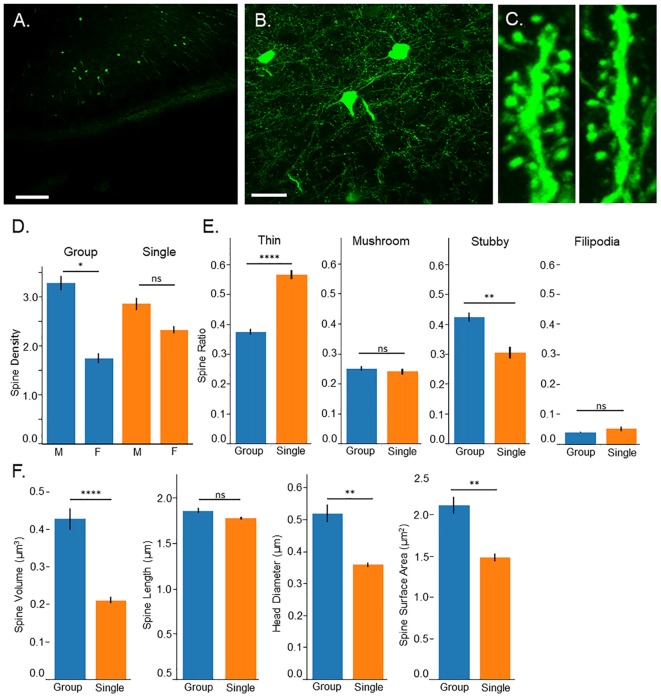
Confocal microscopy. **(A)** 10× image of Thy-1-GFP expression in the prefrontal cortex, pyramidal layer 3, scale bar = 200 μm. **(B)** 60× image of isolated neurons in the prefrontal cortex, pyramidal layer 3, scale bar = 50 μm. **(C)** 3D image of dendritic spines from group housed *(left)* and single housed *(right)* mice. **(D)** Single housed mice show no significant differences in spine density compared to group housed animals; however, a sex difference is found among group housed mice with males having greater spine density. Single housed animals show no significant differences between sexes. **(E)** Single housed mice show a significantly increased ratio of thin spines and significantly fewer stubby spines compared to group housed mice. Single housed and group housed mice show no significant differences on spine classifications of mushroom or filopodia. **(F)** Single housed mice show significantly reduced overall spine volume, reduced head diameter, and reduced spine surface area, showing evidence of immature dendritic spines. **(D)** *Tukey’s Post Hoc*: *p < 0.05; **(E,F)** Main Effect for Housing Condition: *p < 0.05, **p < 0.01, ****p < 0.0001. ns, non-significant.

Images were taken from pyramidal cells in layer 2/3 of the prefrontal cortex (Figures 2A–C). This layer has been shown to be related to several psychiatric disorders in humans and rodents, particularly when spine density and morphology are altered (Pierri et al., 2001; Sasaki et al., 2015; Shrestha et al., 2015). Spines were collected beginning 10 μm from the soma. Spines were analyzed on dendritic arbors ranging from 50 to 140 μm in length. Spine density was assessed as an indicator of neuronal network complexity (Bonhoeffer and Yuste, 2002; Lippman and Dunaevsky, 2005; Harms and Dunaevsky, 2007), with the expectation that single housed mice would have reduced spine density in the prefrontal cortex. Spine morphological characteristics were assessed to indicate spine maturity (Matsuzaki et al., 2001; Bonhoeffer and Yuste, 2002; Hayashi and Majewska, 2005; Tanaka et al., 2008). Data was collected from apical dendrites of the prefrontal cortex, given evidence for the role these apical dendrites play in social adaptation (Burleson et al., 2016). Sex differences were also assessed to control for previous reports that spine density within the prefrontal cortex is influenced by sex (Hajszan et al., 2007; Ren et al., 2012).

Surprisingly, no significant differences were found between group housed and single housed animals on spine density (Main effect for Condition: *F*_(1,128)_ = 0.09, *p* = 0.77; Figure 2D); however, a significant sex difference was found (Main effect for sex: *F*_(1,128)_ = 17.74, *p* = 4.7 × 10^−5^). This sex difference was limited to group housed animals (Interaction between housing condition and sex: *F*_(1,128)_ = 4.20, *p* = 0.043). *Post hoc* tests indicate a significantly higher spine density among group housed males compared to group housed females. Single housed mice show no significant differences between sexes.

While overall spine density did not seem to be affected, spine morphology was, with single housed mice showing indicators of immature spines. Several morphological characteristics were assessed including spine classification, overall spine volume, total extent from the dendritic arbor (length), spine head diameter and spine surface area. When spines undergo LTP, they undergo changes that result in enlarging of the spine which can be quantified as an increase in spine head diameter, increase in overall volume, and increase in spine length (Hayashi and Majewska, 2005; Tanaka et al., 2008). A greater ratio of thin spines compared to mushroom spines can indicate reduced numbers of spines undergoing LTP-induced morphological changes, indicating an immaturity among the spines.

Single housed mice show a significantly higher ratio of thin spines (*F*_(1,128)_ = 34.93, *p* = 2.9 × 10^−8^) and a significantly lower ratio of stubby spines (*F*_(1,128)_ = 9.15, *p* = 0.003; Figure 2E). No significant differences were found between housing conditions on the ratio of mushroom spines or filopodia spines. When observing morphological characteristics, single housed mice show additional signs of immature spines, with significantly reduced overall spine volume and significantly reduced total surface area (*F*_(1,128)_ = 15.48, *p* = 0.0001, *F*_(1,128)_ = 8.47, *p* = 0.004, respectively; Figure 2F). No differences were found in the extent of the spines from the dendrite. Furthermore, single housed mice show significantly reduced spine head diameter (*F*_(1,128)_ = 7.81, *p* = 0.006), suggesting an inability to stabilize plastic changes induced by LTP, which results in an enlarging of the spine head. No sex differences were observed on morphological characteristics and males and females were grouped for morphological analysis. Taken together, these results indicate an immaturity among dendritic spines in the prefrontal cortex of single housed animals.

### Single Housed Mice Exhibit Hypoactivation and Impaired Plasticity in the mPFC

**Figure 3:**
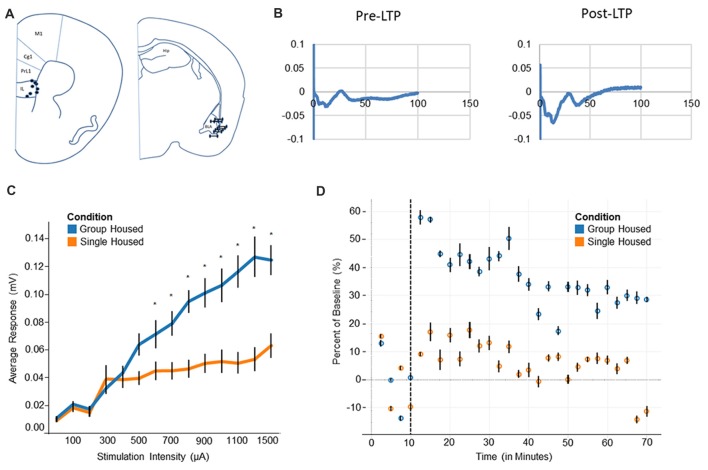
Electrophysiology and long-term potentiation (LTP). **(A)** Electrode placement for medial prefrontal cortex electrode (mPFC) and basolateral amygdala (BLA) indicates all recorded mice fell within the regions of interest. **(B)** Evoked potentials for BLA-mPFC pathway show a negative wave followed by a positive wave. After LTP induction the negative wave becomes markedly increased. **(C)** Input/Output curve shows single housed mice reach a saturation point at roughly 300 μA, compared to 1300 μA among group housed mice. **(D)** Group housed mice show a heightened response after LTP induction, plateauing at a new baseline level towards the end of 1 h. Single housed mice, by contrast, show little response to LTP and decline back to the original baseline level after 1 h. Tukey’s *Post Hoc*: *p < 0.05.

Electrodes were placed in the basolateral amygdala and the infralimbic area of the medial prefrontal cortex (Figure 3A). Locations of the electrodes were verified following recording and any mice with electrodes outside the target regions were not included. Test-pulses were delivered to the amygdala, and responses were recorded from the medial prefrontal cortex. Evoked potentials in the mPFC took on a characteristic negative wave, followed by a positive wave before returning to baseline (Figure 3B). Data were collected and analyzed utilizing the difference between the minimum data point within this negative wave, and the maximum data point within the positive wave. When LTP is induced, this difference was shown to be an effective measure of the change in evoked potentials from baseline (Figure 3B, right). LTP data is displayed as the percent difference from baseline.

Single housed mice show impaired activation of the prefrontal cortex, as evidenced by the input/output response. A significant interaction of housing condition by input was found (*F*_(13,63)_ = 4.79, *p* = 1.0 × 10^−5^; Figure 3C), indicating different responses by housing condition to similar levels of input. *Post hoc* tests reveal single housed mice appear very similar to group housed mice until roughly 600 μA, where group housed animals and single housed animals diverge quite dramatically. Single housed animals reach saturation very early, with increasing stimulation beyond 300 μA inducing little change in neuronal response. Group housed animals, on the other hand, reach a saturation level at roughly 1300 μA (Figure 3C). No effect for sex was found (*F*_(1,5)_ = 1.08, *p* = 0.35). Taken together, this suggests a hypoactivation within the prefrontal cortex of socially isolated animals, which has been associated with many disorders including anxiety, depression, and attention deficits (Matsuo et al., 2003; Zhong et al., 2011; Pezze et al., 2014; van Harmelen et al., 2014), behavioral characteristics of which are found among socially isolated animals.

Socially isolated animals show severely impaired LTP consolidation. Upon induction of LTP, group housed animals show a large change in response, resulting in a sharp difference from baseline (Figure 3D). This response declines over time, while remaining well above the original baseline level. Single housed mice, by contrast, show little change in response after induction of LTP. These animals show a muted response after LTP is induced, declining nearly to baseline at the end of the 1 h period. This suggests an impaired ability to engage in plastic changes, as well as an inability to undergo longer term consolidation.

## Discussion

The above results demonstrate a loss of neuronal adaptability among single housed animals. In addition to the abnormal social behavior, hyperlocomotive behaviors, and some depressive-like behaviors, single housed animals exhibited an inability to undergo stable LTP responses within the prefrontal cortex. Confocal imaging demonstrated a lack of maturity among dendritic spines in this region, which may contribute to the lack of stable LTP responses.

Behavior effects of the mice socially isolated throughout adolescence confirms findings identified by others. Socially isolated mice show depressive-like behaviors, hyperlocomotion, and reduced social behaviors, all of which have been previously documented (Koike et al., 2009; Amiri et al., 2015a; Castillo-Gómez et al., 2017). These behaviors are consistent with several genetic models of autism (Shemesh et al., 2016; Zhou et al., 2016). Social behavior appears impaired in both male and female single housed mice; however, group housed female animals demonstrated an unexpected avoidance behavior. Many studies indicate males and females interact socially in different ways (Walf and Frye, 2008; Clipperton Allen et al., 2010; van den Berg et al., 2015); however, previous studies on socially isolated animals have only used males when testing social behaviors (Koike et al., 2009; Liu et al., 2016). Evidence shows estrous cycles can alter social behavior (Hanson and Hurley, 2012; McHenry et al., 2017). Given that our animals were housed in the same room, it is likely that the estrous cycles were in sync which may have influenced the behavior observed in our female mice. Additionally, consistent with our findings, females have been previously shown to demonstrate reduced spine density in the medial prefrontal cortex compared to males (Hajszan et al., 2007), an area related to social behavior. Although no sex differences were noted on dendritic spine morphology and long-term potentiation in our study, a larger sample size and further testing is required to fully elucidate potential differences between sexes in socially isolated housing conditions.

Depressive-like behavior has also been previously documented in socially isolated mice (Koike et al., 2009). In addition to the depressive-like behaviors seen in our test of the forced swim test, the mice tested show reduced grooming behavior in an open field observation. Previous experiments have described a lack of self-care among socially isolated mice as a possible indicator of depression, which appears to be replicated in our experiments. An alternative explanation is that the socially isolated animals demonstrate an alternate means of coping with the stress of the situation, given that the forced swim test has been shown to test stress coping behaviors (Commons et al., 2017). The increased rest time after subsequent trials may indicate a quicker readiness to abandon this strategy.

The immaturity among dendritic spines correlates with the lack of LTP. It has long been assumed that the enlargement of dendritic spines occurs as a direct result of LTP (Hayashi and Majewska, 2005; Lippman and Dunaevsky, 2005). More recently this has been confirmed by demonstrating that enlargement only occurs at dendritic spines where LTP has been triggered (Tanaka et al., 2008). The lack of LTP found in single housed animals, combined with hypoactivation seen in the input/output curve, suggests even a strong stimulus is insufficient to trigger LTP-like activity. Significantly reduced spine head diameter, as well as the overall size of the spines, suggests the biochemical response to LTP, including protein synthesis and α-amino-3-hydroxy-5-methyl-4-isoxazolepropionic acid receptor (AMPAR) trafficking, does not occur (Matsuzaki et al., 2001; Hayashi and Majewska, 2005; Tanaka et al., 2008).

The observed behaviors among socially isolated mice correspond to those seen in disorders such as autism. In addition, immature spines have been demonstrated in both mouse models of and in human disorders such as autism and fragile-X, where social behavior is highly disrupted (Wei et al., 2012; He and Portera-Cailliau, 2013; Phillips and Pozzo-Miller, 2015). It is not uncommon for these disorders to present with anxiety and depressive behaviors, which can be treated through medications. Current therapeutics also include behavioral therapies, targeting the aberrant behaviors (Ben-Itzchak and Zachor, 2007; Tiura et al., 2017). Our results suggest ability to alter this behavior in adulthood, particularly those related to the prefrontal cortex, may be increasingly difficult since LTP is impaired within this region. This may explain why these behavioral therapies have declining success as the patients get older (Eikeseth et al., 2002; Ben-Itzchak and Zachor, 2007; Tiura et al., 2017).

It has been suggested in cases of autism that spine turnover is higher than normal, leading to an inability to stabilize mature spines (He and Portera-Cailliau, 2013). In the case of single housed mice there may be a disruption of activity dependent development, which has been shown to disrupt spine morphology into adulthood in areas such as the hippocampus (Xing et al., 2016). It is likely that there is mutual contribution of the dendritic spine effects to the behavior, as well as the behavior to the immaturity of the spines (Gipson and Olive, 2017).

Some of the observed effects may be caused by changes affecting either neurotransmission or myelination of the neurons within the prefrontal cortex. Studies have shown that social isolation during adolescence can alter neurotransmission (Haj-Mirzaian et al., 2015; Okada et al., 2015), and can even affect expression of neurotransmitter receptors such as N-Methyl-D-Aspartate (NMDA; Gan et al., 2014; Shimizu et al., 2016). Others have demonstrated that changes in myelination can occur after prolonged periods of isolation in adult mice, and some behaviors can be rescued by increasing this myelination (Liu et al., 2012, 2016). Each of these may contribute to decreased neuronal activation, playing a role in the hypoactivation seen in our experiments.

Critical periods are identified as a period of heightened plasticity, and provide a period of increased modification of neural circuits that lays the framework for behavior in adult animals (Hensch, 2005; Morishita and Hensch, 2008; Erzurumlu and Gaspar, 2012). Isolation during the critical developmental period of adolescence likely disrupts a delicate balance of excitation and inhibition within the prefrontal cortex (Rubenstein, 2010; Yizhar et al., 2011), which has been hypothesized as a cause for dysfunction in autism disorders (Rubenstein and Merzenich, 2003; LeBlanc and Fagiolini, 2011; Kim et al., 2013). The prefrontal cortex undergoes a number of important developmental changes during adolescence, and disruptions to this can result in complex behavioral changes associated with disorders such as autism or schizophrenia (Kolb and Nonneman, 1976; Bossong and Niesink, 2010). Disruptions to the balance of excitatory inputs may result in inappropriate pruning, leading to the loss of critical neuronal connections (Chaudhury et al., 2016; Piochon et al., 2016). This also may lead to reduced responses within this region, or hypoactivation, which has been associated with depression and anxiety behaviors (Matsuo et al., 2003; Zhong et al., 2011; Pezze et al., 2014; van Harmelen et al., 2014). This may explain the immature dendritic spines identified in our experiments, as well as the lack of response in the prefrontal cortex to an aggressive stimulation protocol.

The prefrontal cortex has connections with many brain regions, including the hippocampus (Parent et al., 2010; Spellman et al., 2015), the midbrain (Lee et al., 2014) and other areas of the cortex (Yeterian et al., 2012; Almada et al., 2015; Barredo et al., 2016). The amygdala-medial prefrontal pathway provides an established pathway known to undergo LTP-like changes (Maroun and Richter-Levin, 2003). As research into circuit characteristics of socially isolated mice continues, it is likely that other pathways will be found to be affected, with resulting changes in plasticity. The pathway used in our experiment provided a starting point to assess whether any differences were found.

Future research efforts should focus on identification of the critical developmental periods, recovery of behavioral deficits and delineation of affected pathways. Evidence from this study, as well as others, suggests isolation during the adolescent period permanently affects behavior; however, this has yet to be directly tested. Future research should investigate whether the observed deficits can be recovered through return to group housing conditions. These results would have implications for equivalent behaviors in humans and whether recovery of existing deficits could occur. Current treatments among humans for disorders of social behavior include combinations of behavioral therapies and medications (Chahrour et al., 2016). Our results suggest behavioral recovery may be limited by reduced LTP in the prefrontal cortex. These results would also be relevant in many societies where select populations experience social isolation, which this has implications for their physical and mental health (Tanskanen and Anttila, 2016; Wang et al., 2017; Rico-Uribe et al., 2018).

Recovery of behavioral deficits has been demonstrated among adult animals temporarily isolated (Liu et al., 2012, 2016); however, behavioral recovery among mice reared in isolation has yet to be tested. Efforts to test this may be hampered by increased aggression among these animals after isolation (Ma et al., 2011; Toth et al., 2011, 2012; Tulogdi et al., 2014; Perkeybile and Bales, 2015). Finally, many pathways may be affected by the changes within the prefrontal cortex. Further studies are needed to delineate these pathways to provide a better understanding of the relationship between the underlying neurological changes and the resulting behavioral changes.

The results from our findings suggests limitations of using social isolation in studies where this is necessary such as studies of aggressive animals (Yang et al., 2017; Xu et al., 2018). These studies should approach findings related to spine density or LTP with caution, since social isolation impairs these, particularly in the prefrontal cortex when this isolation occurs during adolescent periods. Our findings should also be considered when single housing animals after implantation of chronic electrodes or fiber optics to ensure results are not impacted by this isolation.

Our findings demonstrate single housed mice have impaired development of the prefrontal cortex, suggesting social interaction during adolescence contributes to the structural and functional maturation of this region, and developmental disruptions underlie behavioral phenotypes in the adult. Our preliminary analyses should motivate further detailed studies of neuronal function and structure in these animals. Such studies might provide insight into the relationship between altered development of brain structures and subsequent behavioral phenotypes with features that correlate with a group of neurodevelopmental disorders, including autism or fragile-X syndromes. These mice may serve as a means to assess development of the prefrontal cortex through critical developmental periods, providing insight into factors contributing to the onset of many psychiatric disorders.

## Author Contributions

WEM, ARM, UH and KAJ contributed conception and design of the study. All authors contributed to acquisition, analysis and interpretation of data. WEM wrote the first draft of the manuscript. All authors contributed to manuscript revision, read and approved the submitted version.

## Conflict of Interest Statement

The authors declare that the research was conducted in the absence of any commercial or financial relationships that could be construed as a potential conflict of interest.

## Footnotes

^1^https://www.jax.org/strain/007788

## References

[B1] Ackman J. B. Crair M. C. (2014). Role of emergent neural activity in visual map development. Curr. Opin. Neurobiol. 24, 166–175. 10.1016/j.conb.2013.11.011 24492092 PMC3957181

[B2] Ago Y. Takahashi K. Nakamura S. Hashimoto H. Baba A. Matsuda T. (2007). Anxiety-like and exploratory behaviors of isolation-reared mice in the staircase test. J. Pharmacol. Sci. 104, 153–158. 10.1254/jphs.fp0070325 17538228

[B3] Almada R. C. Coimbra N. C. Brandão M. L. (2015). Medial prefrontal cortex serotonergic and GABAergic mechanisms modulate the expression of contextual fear: intratelencephalic pathways and differential involvement of cortical subregions. Neuroscience 284, 988–997. 10.1016/j.neuroscience.2014.11.001 25451298

[B4] Amaral D. G. Insausti R. (1992). Retrograde transport of D-[^3^H]-aspartate injected into the monkey amygdaloid complex. Exp. Brain Res. 88, 375–388. 10.1007/bf02259113 1374347

[B5] Amiri S. Amini-Khoei H. Haj-Mirzaian A. Rahimi-Balaei M. Naserzadeh P. Dehpour A.. (2015a). Tropisetron attenuated the anxiogenic effects of social isolation by modulating nitrergic system and mitochondrial function. Biochim. Biophys. Acta 1850, 2464–2475. 10.1016/j.bbagen.2015.09.009 26367080

[B6] Amiri S. Haj-Mirzaian A. Rahimi-Balaei M. Razmi A. Kordjazy N. Shirzadian A.. (2015b). Co-occurrence of anxiety and depressive-like behaviors following adolescent social isolation in male mice; possible role of nitrergic system. Physiol. Behav. 145, 38–44. 10.1016/j.physbeh.2015.03.032 25817356

[B7] Balemans M. C. M. Huibers M. M. H. Eikelenboom N. W. D. Kuipers A. J. van Summeren R. C. J. Pijpers M. M. C. A.. (2010). Reduced exploration, increased anxiety, and altered social behavior: autistic-like features of euchromatin histone methyltransferase 1 heterozygous knockout mice. Behav. Brain Res. 208, 47–55. 10.1016/j.bbr.2009.11.008 19896504

[B8] Barredo J. Verstynen T. D. Badre D. (2016). Organization of cortico-cortical pathways supporting memory retrieval across subregions of the left ventrolateral prefrontal cortex. J. Neurophysiol. 116, 920–937. 10.1152/jn.00157.2016 27281745 PMC5009205

[B9] Ben-Itzchak E. Zachor D. A. (2007). The effects of intellectual functioning and autism severity on outcome of early behavioral intervention for children with autism. Res. Dev. Disabil. 28, 287–303. 10.1016/j.ridd.2006.03.002 16730944

[B10] Bonhoeffer T. Yuste R. (2002). Spine motility. Phenomenology, mechanisms, and function. Neuron 35, 1019–1027. 10.1016/S0896-6273(02)00906-6 12354393

[B11] Bossong M. G. Niesink R. J. M. (2010). Adolescent brain maturation, the endogenous cannabinoid system and the neurobiology of cannabis-induced schizophrenia. Prog. Neurobiol. 92, 370–385. 10.1016/j.pneurobio.2010.06.010 20624444

[B12] Browne C. J. Fletcher P. J. Zeeb F. D. (2016). Responding for a conditioned reinforcer or unconditioned sensory reinforcer in mice: interactions with environmental enrichment, social isolation, and monoamine reuptake inhibitors. Psychopharmacology 233, 983–993. 10.1007/s00213-015-4178-5 26690588

[B13] Burleson C. A. Pedersen R. W. Seddighi S. DeBusk L. E. Burghardt G. M. Cooper M. A. (2016). Social play in juvenile hamsters alters dendritic morphology in the medial prefrontal cortex and attenuates effects of social stress in adulthood. Behav. Neurosci. 130, 437–447. 10.1037/bne0000148 27176563 PMC4961604

[B14] Buschman T. J. Miller E. K. (2014). Goal-direction and top-down control. Philos. Trans. R. Soc. Lond. B. Biol. Sci. 369:20130471. 10.1098/rstb.2013.0471 25267814 PMC4186225

[B15] Cassell M. D. Chittick C. A. Siegel M. A. Wright D. J. (1989). Collateralization of the amygdaloid projections of the rat prelimbic and infralimbic cortices. J. Comp. Neurol. 279, 235–248. 10.1002/cne.902790207 2913068

[B16] Castillo-Gómez E. Pérez-Rando M. Bellés M. Gilabert-Juan J. Llorens J. V. Carceller H.. (2017). Early social isolation stress and perinatal NMDA receptor antagonist treatment induce changes in the structure and neurochemistry of inhibitory neurons of the adult amygdala and prefrontal cortex. eNeuro 4:ENEURO.0034-17.2017. 10.1523/ENEURO.0034-17.2017 28466069 PMC5411163

[B17] Chahrour M. O’Roak B. J. Santini E. Samaco R. C. Kleiman R. J. Manzini M. C. (2016). Current perspectives in autism spectrum disorder: from genes to therapy. J. Neurosci. 36, 11402–11410. 10.1523/JNEUROSCI.2335-16.2016 27911742 PMC5125207

[B19] Chaudhury S. Sharma V. Kumar V. Nag T. C. Wadhwa S. (2016). Activity-dependent synaptic plasticity modulates the critical phase of brain development. Brain Dev. 38, 355–363. 10.1016/j.braindev.2015.10.008 26515724

[B18] Chaudhury D. Walsh J. J. Friedman A. K. Juarez B. Ku S. M. Koo J. W.. (2013). Rapid regulation of depression-related behaviours by control of midbrain dopamine neurons. Nature 493, 532–536. 10.1038/nature11713 23235832 PMC3554860

[B20] Clipperton Allen A. E. Cragg C. L. Wood A. J. Pfaff D. W. Choleris E. (2010). Agonistic behavior in males and females: effects of an estrogen receptor β agonist in gonadectomized and gonadally intact mice. Psychoneuroendocrinology 35, 1008–1022. 10.1016/j.psyneuen.2010.01.002 20129736 PMC2891273

[B21] Commons K. G. Cholanians A. B. Babb J. A. Ehlinger D. G. (2017). The rodent forced swim test measures stress-coping strategy, not depression-like behavior. ACS Chem. Neurosci. 8, 955–960. 10.1021/acschemneuro.7b00042 28287253 PMC5518600

[B22] Crawley J. N. (1985). Exploratory behavior models of anxiety in mice. Neurosci. Biobehav. Rev. 9, 37–44. 10.1016/0149-7634(85)90030-2 2858080

[B23] Crawley J. N. (2007). What’s Wrong With My Mouse?: Behavioral Phenotyping of Transgenic and Knockout Mice. Hoboken, NY: John Wiley & Sons.

[B24] Eikeseth S. Smith T. Jahr E. Eldevik S. (2002). Intensive behavioral treatment at school for 4- to 7-year-old children with autism: a 1-year comparison controlled study. Behav. Modif. 26, 49–68. 10.1177/0145445502026001004 11799654

[B25] Eluvathingal T. J. Chugani H. T. Behen M. E. Juhász C. Muzik O. Maqbool M.. (2006). Abnormal brain connectivity in children after early severe socioemotional deprivation: a diffusion tensor imaging study. Pediatrics 117, 2093–2100. 10.1542/peds.2005-1727 16740852

[B26] Erzurumlu R. S. Gaspar P. (2012). Development and critical period plasticity of the barrel cortex. Eur. J. Neurosci. 35, 1540–1553. 10.1111/j.1460-9568.2012.08075.x 22607000 PMC3359866

[B27] Feng G. Mellor R. H. Bernstein M. Keller-Peck C. Nguyen Q. T. Wallace M.. (2000). Imaging neuronal subsets in transgenic mice expressing multiple spectral variants of GFP. Neuron 28, 41–51. 10.1016/s0896-6273(00)00084-2 11086982

[B28] Fox M. E. Studebaker R. I. Swofford N. J. Wightman R. M. (2015). Stress and drug dependence differentially modulate norepinephrine signaling in animals with varied HPA axis function. Neuropsychopharmacology 40, 1752–1761. 10.1038/npp.2015.23 25601230 PMC4915259

[B29] Frenkel M. Y. Bear M. F. (2004). How monocular deprivation shifts ocular dominance in visual cortex of young mice. Neuron 44, 917–923. 10.1016/j.neuron.2004.12.003 15603735

[B30] Gan J. O. Bowline E. Lourenco F. S. Pickel V. M. (2014). Adolescent social isolation enhances the plasmalemmal density of NMDA NR1 subunits in dendritic spines of principal neurons in the basolateral amygdala of adult mice. Neuroscience 258, 174–183. 10.1016/j.neuroscience.2013.11.003 24231734 PMC3902844

[B31] Gipson C. D. Olive M. F. (2017). Structural and functional plasticity of dendritic spines—root or result of behavior? Genes Brain Behav. 16, 101–117. 10.1111/gbb.12324 27561549 PMC5243184

[B32] Groenewegen H. J. Uylings H. B. (2000). The prefrontal cortex and the integration of sensory, limbic and autonomic information. Prog. Brain Res. 126, 3–28. 10.1016/s0079-6123(00)26003-2 11105636

[B33] Haj-Mirzaian A. Amiri S. Kordjazy N. Rahimi-Balaei M. Haj-Mirzaian A. Marzban H.. (2015). Blockade of NMDA receptors reverses the depressant, but not anxiogenic effect of adolescence social isolation in mice. Eur. J. Pharmacol. 750, 160–166. 10.1016/j.ejphar.2015.01.006 25592321

[B34] Hajszan T. MacLusky N. J. Johansen J. A. Jordan C. L. Leranth C. (2007). Effects of androgens and estradiol on spine synapse formation in the prefrontal cortex of normal and testicular feminization mutant male rats. Endocrinology 148, 1963–1967. 10.1210/en.2006-1626 17317772 PMC2128740

[B35] Hanson J. L. Hurley L. M. (2012). Female presence and estrous state influence mouse ultrasonic courtship vocalizations. PLoS One 7:e40782. 10.1371/journal.pone.0040782 22815817 PMC3399843

[B36] Harada T. Harada C. Parada L. F. (2007). Molecular regulation of visual system development: more than meets the eye. Genes Dev. 21, 367–378. 10.1101/gad.1504307 17322396

[B37] Harms K. J. Dunaevsky A. (2007). Dendritic spine plasticity: looking beyond development. Brain Res. 1184, 65–71. 10.1016/j.brainres.2006.02.094 16600191

[B38] Hayashi Y. Majewska A. K. (2005). Dendritic spine geometry: functional implication and regulation. Neuron 46, 529–532. 10.1016/j.neuron.2005.05.006 15944122

[B39] He C. X. Portera-Cailliau C. (2013). The trouble with spines in fragile X syndrome: density, maturity and plasticity. Neuroscience 251, 120–128. 10.1016/j.neuroscience.2012.03.049 22522472 PMC3422423

[B40] Hensch T. K. (2005). Critical period plasticity in local cortical circuits. Nat. Rev. Neurosci. 6, 877–888. 10.1038/nrn1787 16261181

[B41] Keifer O. P. Hurt R. C. Gutman D. A. Keilholz S. D. Gourley S. L. Ressler K. J. (2015). Voxel-based morphometry predicts shifts in dendritic spine density and morphology with auditory fear conditioning. Nat. Commun. 6:7582. 10.1038/ncomms8582 26151911 PMC4506522

[B42] Kim H. Gibboni R. Kirkhart C. Bao S. (2013). Impaired critical period plasticity in primary auditory cortex of fragile X model mice. J. Neurosci. 33, 15686–15692. 10.1523/JNEUROSCI.3246-12.2013 24089476 PMC3787494

[B43] Koike H. Ibi D. Mizoguchi H. Nagai T. Nitta A. Takuma K.. (2009). Behavioral abnormality and pharmacologic response in social isolation-reared mice. Behav. Brain Res. 202, 114–121. 10.1016/j.bbr.2009.03.028 19447287

[B44] Kolb B. Nonneman A. J. (1976). Functional development of prefrontal cortex in rats continues into adolescence. Science 193, 335–336. 10.1126/science.935872 935872

[B45] Kumar S. Black S. J. Hultman R. Szabo S. T. DeMaio K. D. Du J.. (2013). Cortical control of affective networks. J. Neurosci. 33, 1116–1129. 10.1523/JNEUROSCI.0092-12.2013 23325249 PMC3711588

[B46] Laird P. W. Zijderveld A. Linders K. Rudnicki M. A. Jaenisch R. Berns A. (1991). Simplified mammalian DNA isolation procedure. Nucleic Acids Res. 19:4293. 10.1093/nar/19.15.4293 1870982 PMC328579

[B47] LeBlanc J. J. Fagiolini M. (2011). Autism: a “critical period” disorder? Neural Plast. 2011:921680. 10.1155/2011/921680 21826280 PMC3150222

[B48] Lee A. T. Vogt D. Rubenstein J. L. Sohal V. S. (2014). A class of GABAergic neurons in the prefrontal cortex sends long-range projections to the nucleus accumbens and elicits acute avoidance behavior. J. Neurosci. 34, 11519–11525. 10.1523/JNEUROSCI.1157-14.2014 25164650 PMC4145166

[B49] Lippman J. Dunaevsky A. (2005). Dendritic spine morphogenesis and plasticity. J. Neurobiol. 64, 47–57. 10.1002/neu.20149 15884005

[B50] Liu J. Dietz K. DeLoyht J. M. Pedre X. Kelkar D. Kaur J.. (2012). Impaired adult myelination in the prefrontal cortex of socially isolated mice. Nat. Neurosci. 15, 1621–1623. 10.1038/nn.3263 23143512 PMC3729624

[B51] Liu J. Dupree J. L. Gacias M. Frawley R. Sikder T. Naik P.. (2016). Clemastine enhances myelination in the prefrontal cortex and rescues behavioral changes in socially isolated mice. J. Neurosci. 36, 957–962. 10.1523/JNEUROSCI.3608-15.2016 26791223 PMC4719024

[B52] Ma X. Jiang D. Jiang W. Wang F. Jia M. Wu J.. (2011). Social isolation-induced aggression potentiates anxiety and depressive-like behavior in male mice subjected to unpredictable chronic mild stress. PloS One 6:e20955. 10.1371/journal.pone.0020955 21698062 PMC3117867

[B53] Maroun M. Richter-Levin G. (2003). Exposure to acute stress blocks the induction of long-term potentiation of the amygdala-prefrontal cortex pathway *in vivo*. J. Neurosci. 23, 4406–4409. 10.1523/JNEUROSCI.23-11-04406.2003 12805280 PMC6740777

[B54] Matsuo K. Taneichi K. Matsumoto A. Ohtani T. Yamasue H. Sakano Y.. (2003). Hypoactivation of the prefrontal cortex during verbal fluency test in PTSD: a near-infrared spectroscopy study. Psychiatry Res. 124, 1–10. 10.1016/s0925-4927(03)00093-3 14511791

[B55] Matsuzaki M. Ellis-Davies G. C. Nemoto T. Miyashita Y. Iino M. Kasai H. (2001). Dendritic spine geometry is critical for AMPA receptor expression in hippocampal CA1 pyramidal neurons. Nat. Neurosci. 4, 1086–1092. 10.1038/nn736 11687814 PMC4229049

[B56] McHenry J. A. Otis J. M. Rossi M. A. Robinson J. E. Kosyk O. Miller N. W.. (2017). Hormonal gain control of a medial preoptic area social reward circuit. Nat. Neurosci. 20, 449–458. 10.1038/nn.4487 28135243 PMC5735833

[B57] Miller E. K. (2000). The prefrontal cortex and cognitive control. Nat. Rev. Neurosci. 1, 59–65. 10.1038/35036228 11252769

[B58] Morishita H. Hensch T. K. (2008). Critical period revisited: impact on vision. Curr. Opin. Neurobiol. 18, 101–107. 10.1016/j.conb.2008.05.009 18534841

[B59] Okada R. Fujiwara H. Mizuki D. Araki R. Yabe T. Matsumoto K. (2015). Involvement of dopaminergic and cholinergic systems in social isolation-induced deficits in social affiliation and conditional fear memory in mice. Neuroscience 299, 134–145. 10.1016/j.neuroscience.2015.04.064 25943484

[B60] Parent M. A. Wang L. Su J. Netoff T. Yuan L.-L. (2010). Identification of the hippocampal input to medial prefrontal cortex *in vitro*. Cereb. Cortex 20, 393–403. 10.1093/cercor/bhp108 19515741 PMC2803736

[B61] Perkeybile A. M. Bales K. L. (2015). Early rearing experience is related to altered aggression and vasopressin production following chronic social isolation in the prairie vole. Behav. Brain Res. 283, 37–46. 10.1016/j.bbr.2015.01.025 25623420 PMC4351180

[B62] Pezze M. McGarrity S. Mason R. Fone K. C. Bast T. (2014). Too little and too much: hypoactivation and disinhibition of medial prefrontal cortex cause attentional deficits. J. Neurosci. 34, 7931–7946. 10.1523/JNEUROSCI.3450-13.2014 24899715 PMC4044251

[B63] Phillips M. Pozzo-Miller L. (2015). Dendritic spine dysgenesis in autism related disorders. Neurosci. Lett. 601, 30–40. 10.1016/j.neulet.2015.01.011 25578949 PMC4496332

[B64] Pierri J. N. Volk C. L. Auh S. Sampson A. Lewis D. A. (2001). Decreased somal size of deep layer 3 pyramidal neurons in the prefrontal cortex of subjects with schizophrenia. Arch. Gen. Psychiatry 58, 466–473. 10.1001/archpsyc.58.5.466 11343526

[B65] Piochon C. Kano M. Hansel C. (2016). LTD-like molecular pathways in developmental synaptic pruning. Nat. Neurosci. 19, 1299–1310. 10.1038/nn.4389 27669991 PMC5070480

[B66] Ren W.-W. Liu Y. Li B.-M. (2012). Stimulation of α_2A_-adrenoceptors promotes the maturation of dendritic spines in cultured neurons of the medial prefrontal cortex. Mol. Cell. Neurosci. 49, 205–216. 10.1016/j.mcn.2011.10.001 22015717

[B67] Reser J. E. (2014). Solitary mammals provide an animal model for autism spectrum disorders. J. Comp. Psychol. 128, 99–113. 10.1037/a0034519 24188618

[B68] Rico-Uribe L. A. Caballero F. F. Martín-María N. Cabello M. Ayuso-Mateos J. L. Miret M. (2018). Association of loneliness with all-cause mortality: a meta-analysis. PLoS One 13:e0190033. 10.1371/journal.pone.0190033 29300743 PMC5754055

[B69] Rubenstein J. L. R. (2010). Three hypotheses for developmental defects that may underlie some forms of autism spectrum disorder. Curr. Opin. Neurol. 23, 118–123. 10.1097/WCO.0b013e328336eb13 20087182

[B70] Rubenstein J. L. R. Merzenich M. M. (2003). Model of autism: increased ratio of excitation/inhibition in key neural systems. Genes Brain Behav. 2, 255–267. 10.1034/j.1601-183x.2003.00037.x 14606691 PMC6748642

[B71] Saitoh A. Ohashi M. Suzuki S. Tsukagoshi M. Sugiyama A. Yamada M.. (2014). Activation of the prelimbic medial prefrontal cortex induces anxiety-like behaviors via N-Methyl-D-aspartate receptor-mediated glutamatergic neurotransmission in mice. J. Neurosci. Res. 92, 1044–1053. 10.1002/jnr.23391 24752881

[B72] Sasaki T. Aoi H. Oga T. Fujita I. Ichinohe N. (2015). Postnatal development of dendritic structure of layer III pyramidal neurons in the medial prefrontal cortex of marmoset. Brain Struct. Funct. 220, 3245–3258. 10.1007/s00429-014-0853-2 25064470

[B73] Séjourné J. Llaneza D. Kuti O. J. Page D. T. (2015). Social behavioral deficits coincide with the onset of seizure susceptibility in mice lacking serotonin receptor 2c. PLoS One 10:e0136494. 10.1371/journal.pone.0136494 26308619 PMC4550412

[B74] Shemesh Y. Forkosh O. Mahn M. Anpilov S. Sztainberg Y. Manashirov S.. (2016). Ucn3 and CRF-R2 in the medial amygdala regulate complex social dynamics. Nat. Neurosci. 19, 1489–1496. 10.1038/nn.4346 27428651

[B75] Shimizu K. Kurosawa N. Seki K. (2016). The role of the AMPA receptor and 5-HT_3_ receptor on aggressive behavior and depressive-like symptoms in chronic social isolation-reared mice. Physiol. Behav. 153, 70–83. 10.1016/j.physbeh.2015.10.026 26522741

[B76] Shrestha P. Mousa A. Heintz N. (2015). Layer 2/3 pyramidal cells in the medial prefrontal cortex moderate stress induced depressive behaviors. Elife 4:e08752. 10.7554/eLife.08752 26371510 PMC4566133

[B77] Spellman T. Rigotti M. Ahmari S. E. Fusi S. Gogos J. A. Gordon J. A. (2015). Hippocampal-prefrontal input supports spatial encoding in working memory. Nature 522, 309–314. 10.1038/nature14445 26053122 PMC4505751

[B78] Suzuki S. Saitoh A. Ohashi M. Yamada M. Oka J.-I. Yamada M. (2016). The infralimbic and prelimbic medial prefrontal cortices have differential functions in the expression of anxiety-like behaviors in mice. Behav. Brain Res. 304, 120–124. 10.1016/j.bbr.2016.01.044 26802727

[B79] Tanaka J. I. Horiike Y. Matsuzaki M. Miyazaki T. Ellis-Davies G. C. R. Kasai H. (2008). Protein synthesis and neurotrophin-dependent structural plasticity of single dendritic spines. Science 319, 1683–1687. 10.1126/science.1152864 18309046 PMC4218863

[B80] Tanskanen J. Anttila T. (2016). A prospective study of social isolation, loneliness, and mortality in finland. Am. J. Public Health 106, 2042–2048. 10.2105/ajph.2016.303431 27631736 PMC5055788

[B81] Tiura M. Kim J. Detmers D. Baldi H. (2017). Predictors of longitudinal ABA treatment outcomes for children with autism: a growth curve analysis. Res. Dev. Disabil. 70, 185–197. 10.1016/j.ridd.2017.09.008 28963874

[B82] Toth M. Mikics E. Tulogdi A. Aliczki M. Haller J. (2011). Post-weaning social isolation induces abnormal forms of aggression in conjunction with increased glucocorticoid and autonomic stress responses. Horm. Behav. 60, 28–36. 10.1016/j.yhbeh.2011.02.003 21316368

[B83] Toth M. Tulogdi A. Biro L. Soros P. Mikics E. Haller J. (2012). The neural background of hyper-emotional aggression induced by post-weaning social isolation. Behav. Brain Res. 233, 120–129. 10.1016/j.bbr.2012.04.025 22548916

[B84] Tseng K. Y. O’Donnell P. (2007). Dopamine modulation of prefrontal cortical interneurons changes during adolescence. Cereb. Cortex 17, 1235–1240. 10.1093/cercor/bhl034 16818475 PMC2204087

[B85] Tulogdi A. Tóth M. Barsvári B. Biró L. Mikics E. Haller J. (2014). Effects of resocialization on post-weaning social isolation-induced abnormal aggression and social deficits in rats. Dev. Psychobiol. 56, 49–57. 10.1002/dev.21090 23168609

[B86] Ueno H. Suemitsu S. Murakami S. Kitamura N. Wani K. Okamoto M.. (2017). Region-specific impairments in parvalbumin interneurons in social isolation-reared mice. Neuroscience 359, 196–208. 10.1016/j.neuroscience.2017.07.016 28723388

[B87] van den Berg W. E. Lamballais S. Kushner S. A. (2015). Sex-specific mechanism of social hierarchy in mice. Neuropsychopharmacology 40, 1364–1372. 10.1038/npp.2014.319 25469681 PMC4397394

[B88] van Harmelen A. L. van Tol M. J. Dalgleish T. van der Wee N. J. A. Veltman D. J. Aleman A.. (2014). Hypoactive medial prefrontal cortex functioning in adults reporting childhood emotional maltreatment. Soc. Cogn. Affect. Neurosci. 9, 2026–2033. 10.1093/scan/nsu008 24493840 PMC4249477

[B89] Vandenberg A. Piekarski D. J. Caporale N. Munoz-Cuevas F. J. Wilbrecht L. (2015). Adolescent maturation of inhibitory inputs onto cingulate cortex neurons is cell-type specific and TrkB dependent. Front. Neural Circuits 9:5. 10.3389/fncir.2015.00005 25762898 PMC4329800

[B90] Vanderschuren L. J. M. J. Achterberg E. J. M. Trezza V. (2016). The neurobiology of social play and its rewarding value in rats. Neurosci. Biobehav. Rev. 70, 86–105. 10.1016/j.neubiorev.2016.07.025 27587003 PMC5074863

[B91] Vanderschuren L. J. Niesink R. J. Van Ree J. M. (1997). The neurobiology of social play behavior in rats. Neurosci. Biobehav. Rev. 21, 309–326. 10.1016/s0149-7634(96)00020-6 9168267

[B92] Walf A. A. Frye C. A. (2008). Conjugated equine estrogen enhances rats’ cognitive, anxiety and social behavior. Neuroreport 19, 789–792. 10.1097/wnr.0b013e3282fe209c 18418258 PMC2572821

[B93] Wang J. Lloyd-Evans B. Giacco D. Forsyth R. Nebo C. Mann F.. (2017). Social isolation in mental health: a conceptual and methodological review. Soc. Psychiatry Psychiatr. Epidemiol. 52, 1451–1461. 10.1007/s00127-017-1446-1 29080941 PMC5702385

[B94] Wei H. Dobkin C. Sheikh A. M. Malik M. Brown W. T. Li X. (2012). The therapeutic effect of memantine through the stimulation of synapse formation and dendritic spine maturation in autism and fragile X syndrome. PloS One 7:e36981. 10.1371/journal.pone.0036981 22615862 PMC3352866

[B95] Wöhr M. (2015). Effect of social odor context on the emission of isolation-induced ultrasonic vocalizations in the BTBR T+tf/J mouse model for autism. Front. Neurosci. 9:73. 10.3389/fnins.2015.00073 25852455 PMC4364166

[B96] Xing B. Li Y. C. Gao W. J. (2016). GSK3β hyperactivity during an early critical period impairs prefrontal synaptic plasticity and induces lasting deficits in spine morphology and working memory. Neuropsychopharmacology 41, 3003–3015. 10.1038/npp.2016.110 27353310 PMC5101547

[B97] Xu X. M. Chi Q. S. Cao J. Zhao Z. J. (2018). The effect of aggression I: the increases of metabolic cost and mobilization of fat reserves in male striped hamsters. Horm. Behav. 98, 55–62. 10.1016/j.yhbeh.2017.12.015 29288636

[B98] Yang M. Silverman J. L. Crawley J. N. (2011). Automated three-chambered social approach task for mice. Curr. Protoc. Neurosci. Chapter 8:Unit 8.26. 10.1002/0471142301.ns0826s56 21732314 PMC4904775

[B99] Yang T. Yang C. F. Chizari M. D. Maheswaranathan N. Burke K. J. Borius M.. (2017). Social control of hypothalamus-mediated male aggression. Neuron 95, 955.e4–970.e4. 10.1016/j.neuron.2017.06.046 28757304 PMC5648542

[B100] Yeterian E. H. Pandya D. N. Tomaiuolo F. Petrides M. (2012). The cortical connectivity of the prefrontal cortex in the monkey brain. Cortex 48, 58–81. 10.1016/j.cortex.2011.03.004 21481342 PMC3161133

[B101] Yizhar O. Fenno L. E. Prigge M. Schneider F. Davidson T. J. O’Shea D. J.. (2011). Neocortical excitation/inhibition balance in information processing and social dysfunction. Nature 477, 171–178. 10.1038/nature10360 21796121 PMC4155501

[B102] Zhong M. Wang X. Xiao J. Yi J. Zhu X. Liao J.. (2011). Amygdala hyperactivation and prefrontal hypoactivation in subjects with cognitive vulnerability to depression. Biol. Psychol. 88, 233–242. 10.1016/j.biopsycho.2011.08.007 21878364

[B103] Zhou Y. Kaiser T. Monteiro P. Zhang X. Van der Goes M. S. Wang D.. (2016). Mice with Shank3 mutations associated with ASD and schizophrenia display both shared and distinct defects. Neuron 89, 147–162. 10.1016/j.neuron.2015.11.023 26687841 PMC4754122

[B104] Zlatković J. Bernardi R. E. Filipović D. (2014). Protective effect of Hsp70i against chronic social isolation stress in the rat hippocampus. J. Neural. Transm. 121, 3–14. 10.1007/s00702-013-1066-1 23851625

